# Comparison of wavelet and correlation indices of cerebral autoregulation in a pediatric swine model of cardiac arrest

**DOI:** 10.1038/s41598-020-62435-8

**Published:** 2020-04-03

**Authors:** Xiuyun Liu, Xiao Hu, Ken M. Brady, Raymond Koehler, Peter Smielewski, Marek Czosnyka, Joseph Donnelly, Jennifer K. Lee

**Affiliations:** 1 Department of Anesthesiology and Critical Care Medicine, School of Medicine, Johns Hopkins University, Baltimore, MD USA; 2 Department of Physiological Nursing, University of California, San Francisco, CA USA; 3 Department of Neurosurgery, School of Medicine, University of California, Los Angeles, CA USA; 4 Department of Neurological Surgery, University of California, San Francisco, CA USA; 5 Institute of Computational Health Sciences, University of California, San Francisco, CA USA; 6 Northwestern University, Ann & Robert H. Lurie Children’s Hospital of Chicago, Department of Anesthesiology, Chicago, IL USA; 7 Brain Physics Laboratory, Department of Clinical Neurosciences, Addenbrooke’s Hospital, University of Cambridge, Cambridge, UK; 8 Department of Anaesthesiology, University of Auckland, Auckland, New Zealand; 9 Department of Anesthesiology and Critical Care Medicine, Division of Pediatric Anesthesiology, Johns Hopkins University, Baltimore, MD USA; 10 Institute of Electronic Systems, Warsaw University of Technology, Warsaw, Poland

**Keywords:** Neuroscience, Medical research, Neurology

## Abstract

Existing cerebrovascular blood pressure autoregulation metrics have not been translated to clinical care for pediatric cardiac arrest, in part because signal noise causes high index time-variability. We tested whether a wavelet method that uses near-infrared spectroscopy (NIRS) or intracranial pressure (ICP) decreases index variability compared to that of commonly used correlation indices. We also compared whether the methods identify the optimal arterial blood pressure (ABPopt) and lower limit of autoregulation (LLA). 68 piglets were randomized to cardiac arrest or sham procedure with continuous monitoring of cerebral blood flow using laser Doppler, NIRS and ICP. The arterial blood pressure (ABP) was gradually reduced until it dropped to below the LLA. Several autoregulation indices were calculated using correlation and wavelet methods, including the pressure reactivity index (PRx and wPRx), cerebral oximetry index (COx and wCOx), and hemoglobin volume index (HVx and wHVx). Wavelet methodology had less index variability with smaller standard deviations. Both wavelet and correlation methods distinguished functional autoregulation (ABP above LLA) from dysfunctional autoregulation (ABP below the LLA). Both wavelet and correlation methods also identified ABPopt with high agreement. Thus, wavelet methodology using NIRS may offer an accurate vasoreactivity monitoring method with reduced signal noise after pediatric cardiac arrest.

## Introduction

Cerebrovascular autoregulation constrains cerebral blood flow across fluctuations in cerebral perfusion pressure (CPP). It is mediated by vasoreactivity with changes in cerebrovascular resistance. Autoregulation may become dysfunctional after cardiac arrest, traumatic brain injury, and elevated intracranial pressure (ICP)^1–6^ with shifts in the limits of blood pressure autoregulation. Methods to identify and target the blood pressure range that supports autoregulation could reduce secondary neurologic injury.

The optimal mean arterial blood pressure (ABPopt) at which autoregulatory vasoreactivity is most robust varies after pediatric hypoxic brain injury^4,7–9^. Targeting the optimal CPP may improve neurologic outcomes after adult traumatic brain injury^5,10^. However, the ABPopt must be used after pediatric cardiac arrest because invasive ICP monitoring is not routinely performed in this population. Maintaining blood pressure close to ABPopt is associated with less neurologic injury in infants and children at risk of hypoxic brain injury, including those resuscitated from cardiac arrest^4,11^ and those with hypoxic-ischemic encephalopathy^7–9^, prematurity^12^, or moyamoya vasculopathy^13^.

Near-infrared spectroscopy (NIRS) enables continuous autoregulation monitoring in the frontal cortex through a common method which uses low pass filters and correlation coefficients between perfusion pressure and surrogate measures of cerebral blood flow or cerebral blood volume^5,14^. The correlation method is frequently used in traumatic brain injury^5^ and cardiopulmonary bypass^15^ research. Although we found that correlation between a NIRS-derived cerebral blood volume measure and ABP was associated with outcome after pediatric cardiac arrest^4^, we could not predict fine neurologic deficits with this method.

Here, we sought a new metric for autoregulation and vasoreactivity monitoring in pediatric hypoxic brain injury. Our group validated a wavelet semblance method between ABP and ICP^16^ that was better able to identify the blood pressure lower limit of autoregulation (LLA) in piglets with intracranial hypertension than a commonly used, ICP-based, correlation metric called the pressure reactivity index (PRx)^17^. Moreover, the wavelet method more consistently identified optimal CPP and better predicted mortality than the correlation-based PRx in adult brain trauma^10^. The improved ability to identify optimal CPP using wavelet methodology may be related to lower index time-variability (‘variability’ will be used for the remaining part of the article) with less signal noise. In the current study, we compared wavelet and correlation metrics in a piglet model of cardiac arrest. Several indices for autoregulation assessment were calculated using correlation and wavelet methods, including the PRx and wavelet PRx (wPRx), cerebral oximetry index (COx) and wavelet Cox (wCOx), hemoglobin volume index (HVx) and wavelet wHVx (wHVx). We hypothesized that the wavelet method would reduce autoregulation index variability compared to the correlation method and that wavelet indices can distinguish functional from dysfunctional autoregulation in the developing brain after cardiac arrest. We also compared ABPopt values identified by wavelet to those from correlation metrics.

## Results

One piglet was excluded from the study because its recording period was too short. Data were available for wavelet analysis from 35 piglets resuscitated from cardiac arrest and 33 sham piglets^14,18,19^. The mean ABP LLA was 49 mmHg (SD: 9) in cardiac arrest piglets and 45 mmHg (SD: 10) in sham piglets. The piglets’ mean ICP was ≤15 mmHg^14,18,19^.

### Distinguishing ABP above from ABP below the LLA

**Figure 1:**
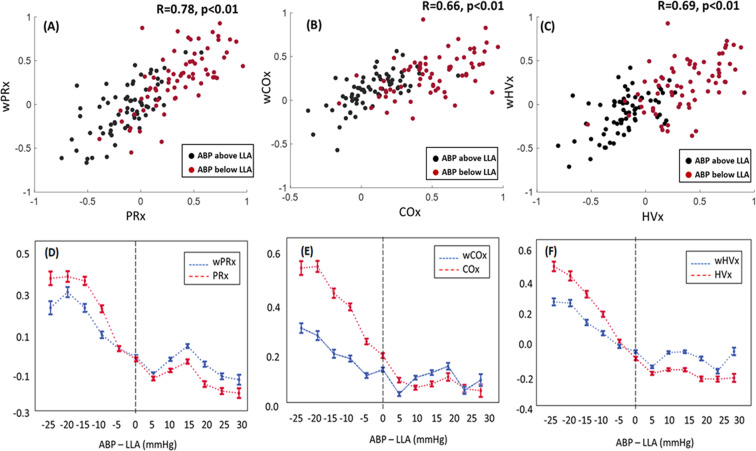
Both wavelet and correlation indices increased while mean arterial blood pressure (ABP) decreased below the lower limit of autoregulation (LLA). (**A–C**) In paired comparisons among 68 piglets, the wavelet autoregulation indices (wPRx, wCOx, wHVx) and correlation indices (PRx, COx and HVx) correlated with each other. Each piglet provided one index value averaged from ABP above the LLA and one index from ABP below the LLA, thereby generating 136 data points per graph. The black dots are index values when ABP exceeded the LLA. The red dots are index values when ABP was below the LLA. (**D–F**) Graphical depiction of the indices across changes in blood pressure. Each piglet’s ABP LLA is centered at zero on the x-axis (dashed line) to show the wavelet method (blue lines) and correlation method (red lines) responses to changes in blood pressure. Data are shown as mean ± standard error of the mean.

Wavelet indices and their respective correlation indices were highly correlated (r = 0.78, *p* < 0.001 for PRx and wPRx; r = 0.66, *p* < 0.001 for COx and wCOx; and r = 0.69, *p* = 0.001 for HVx and wHVx; Fig. 1A–C). Both wavelet and correlation indices were higher overall when ABP was below the LLA than when ABP was above the LLA (Fig. 1D–F). All the correlation indices became positive and approached +1 as blood pressure deviated farther below the LLA during induced hypotension. The wavelet indices also increased as ABP decreased below the LLA but to a lesser degree than that observed with the correlation indices. Mean values for PRx, wPRx, COx, wCOx, HVx, and wHVx were each significantly lower when ABP was above the LLA than when ABP was below the LLA in paired comparisons (*p* < 0.001 for each index; Supplemental Figure S1).

All indices discriminated ABP above the LLA from ABP below the LLA (*p* < 0.001 for all indices’ ROC areas under the curve [AUC]; Table 1). The AUC for distinguishing ABP above versus that below the LLA for HVx was higher than for wHVx (*p* = 0.003). The AUCs were not different between COx and wCOx (*p* = 0.11) or between PRx and wPRx (p = 0.41). The index cut-offs with maximal sensitivity and specificity for distinguishing ABP above from that below the LLA are shown in Table 1.

**Table 1:** Areas under the receiver operator characteristic curve for each index’s ability to distinguish mean arterial blood pressure (ABP) above versus ABP below the lower limit of autoregulation.

Index	n	AUC	p-value	Cut-off index value	Sensitivity at cut-off index value (95% CI)	Specificity at cut-off index value (95% CI)
PRx^a^	68	0.91	<0.001	0.15	0.93(0.84,0.98)	0.84(0.73,0.92)
wPRx^a^	68	0.90	<0.001	0.24	0.88(0.78,0.95)	0.78(0.66,0.87)
COx^a^	68	0.89	<0.001	0.29	0.84 (0.73, 0.92)	0.79 (0.68, 0.88)
wCOx^a^	68	0.84	<0.001	0.26	0.87 (0.76, 0.94)	0.71 (0.58, 0.81)
HVx*	68	0.95	<0.001	0.11	0.93(0.84, 0.98)	0.85 (0.75, 0.93)
wHVx*	68	0.87	<0.001	0.19	0.90 (0.80, 0.96)	0.75 (0.63, 0.85)

AUC, area under the curve; CI, confidence interval. wPRx, wavelet pressure reactivity index. wCOX, wavelet cerebral oximetry index. wHVx, wavelet hemoglobin volume index.

^a^The AUC did not differ between PRx and wPRx (p = 0.41) or between COx and wCOx (p = 0.11).

*p = 0.003 for AUC for HVx vs. AUC for wHVx.

### Wavelet methodology reduces index variability

**Figure 2:**
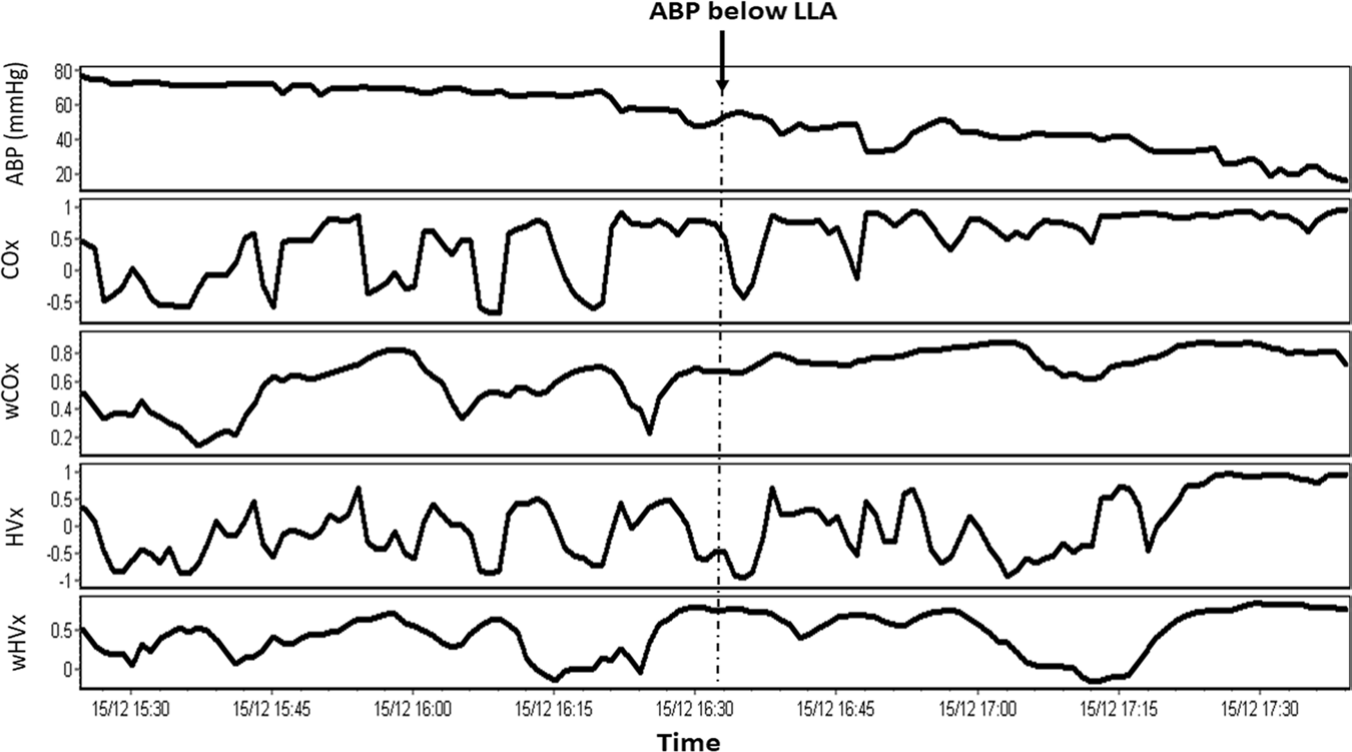
An example piglet’s mean arterial blood pressure (ABP), cerebral oximetry index (COx), wavelet COx (wCOx), hemoglobin volume index (HVx), and wavelet HVx (wHVx) across time. Variability is visually lower in the wavelet indices than in the correlation indices. The time point where the piglet’s ABP crossed to below its lower limit of autoregulation (LLA) is noted.

The wavelet methodology decreased variability in the index signals compared to those calculated by the correlation method (Fig. 2). When ABP was above the LLA, wCOx had a standard SD of 0.31 (±0.09) and COx had a higher SD of 0.38 (±0.10; *p* < 0.001). The wHVx index had an SD of 0.30 (±0.09), whereas HVx had a higher SD of 0.40 (±0.12; *p* < 0.001). The wPRx index had an SD of 0.29 (±0.09), whereas PRx had a higher SD of 0.40 (±0.12; *p* < 0.001).

### Optimal mean arterial blood pressure

**Figure 3:**
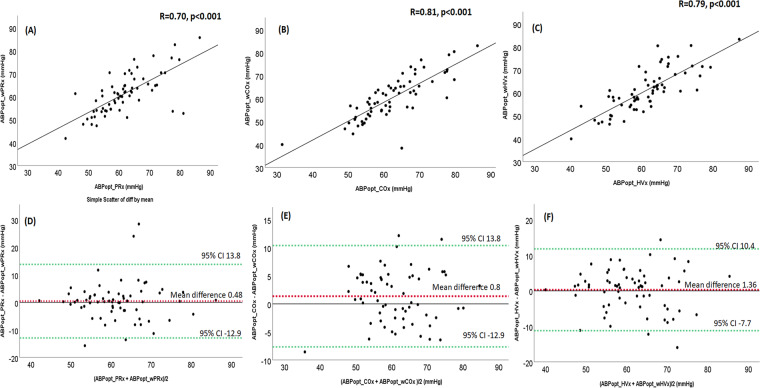
The optimal mean arterial blood pressure (ABPopt) derived by the multi-window method from the wavelet indices correlated with the multi-window ABPopt from the correlation indices. (**A**) PRx and wPRx (n = 66) and (**B**) COx and wCOx (n = 66), (**C**) HVx and wHVx (n = 66). (**D–F)** Bland Altman plots showed overall agreement between ABP_OPT_ from (**D)** PRx and wPRx, (**E**) Cox and wCOx and (**F**) HVx and wHVx.

When ABP was above the LLA, the ABPopt values were identified in 66/68 (97%) piglets with PRx, wPRx, COx, wCOx, HVx, and wHVx using the multi-window method. The COx and wPRx identified ABP_OPT_ in 67/68 (99%) piglets. Among piglets with ABPopt identified by both wavelet and the corresponding correlation index, the ABPopt values correlated (r = 0.70, *p* < 0.001 for PRx and wPRx; r = 0.81, *p* < 0.001 for COx and wCOx; and r = 0.79, *p* < 0.001 for HVx and wHVx; Fig. 3A–C). The wavelet and corresponding correlation indices had good agreement (Fig. 3D–F).

**Figure 4:**
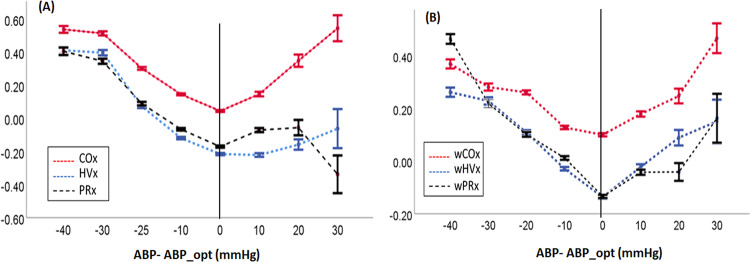
Identifying the optimal mean arterial blood pressure (ABPopt) at the index curve’s vertex. Each piglet’s ABPopt from the multi-window method is centered at zero (solid black line). (**A**) The correlation indices became increasingly positive as blood pressure decreased below ABPopt. However, the PRx and HVx did not increase overall as blood pressure rose above the ABPopt. As a result, the correlation indices did not have clear U-shapes and vertices for ABPopt were not visually apparent for HVx and PRx (n = 66 piglets with an identified ABPopt from PRx and HVx; n = 67 for COx). (**B**) The wavelet indices showed a clearer U-shape with progressively higher values as blood pressure deviated lower or higher than ABPopt. This clearer U-shape enables easier visual identification of ABPopt at the vertex (n = 66 piglets with an identified ABPopt from wHVx and wCOx; n = 67 for wPRx). Data are shown as means ± standard error of the mean.

Visual inspection of the data showed that the correlation indices increased as blood pressure deviated below ABPopt, but they did not consistently increase with blood pressure above the ABPopt for HVx and PRx (Fig. 4A). As a result, the correlation indices did not generate U-shaped curves, and a vertex for ABPopt was not visually apparent in the pooled data for HVx and PRx. In contrast, all of the wavelet indices generated clearer U-shaped curves with apparent ABPopt at the vertices (Fig. 4B).

## Discussion

We compared wavelet autoregulation monitoring methodology to a commonly used correlation method to assess cerebral autoregulation and identify ABPopt in piglets resuscitated from cardiac arrest. Laser Doppler flowmetry provided a standard measure of autoregulatory function to identify the individual LLA in each piglet. This allowed us to separate the wavelet and correlation index data into functional autoregulation (ABP above LLA) or dysfunctional autoregulation (ABP below LLA). All wavelet and correlation indices distinguished functional from dysfunctional autoregulation. The wavelet method reduced index variability with a significant decrease in SD for wHVx, wCOx, wPRx relative to their respective correlation indices. Additionally, agreement between ABPopt values between wavelet and correlation indices was high. The pooled wavelet index curves had more apparent U-shapes with ABPopt at the vertex than did the correlation index curves. Thus, our preclinical data indicate that wavelet NIRS indices may be accurate methods to monitor autoregulation with lower signal variation than commonly used correlation indices^2–4^ in pediatric cardiac arrest.

Multiple autoregulation metrics have been tested clinically after cardiac arrest, including correlation between blood pressure and NIRS surrogate measures of cerebral blood flow or cerebral blood volume^2–4^. Poorer autoregulatory function is associated with higher risk of mortality and severe disability, such as the requirement for tracheostomy or gastrostomy tube, in adults and children after cardiac arrest^2,4^. But the ability to distinguish fine neurologic deficits remains limited with these methods.

Here, we provide preclinical validation of wavelet NIRS autoregulation monitoring against a standard laser Doppler flow measure of cerebral blood flow in the developing brain at term gestation. Higher wavelet semblance with lower phase shift between ABP and NIRS regional cortical oxygen saturation (rSO_2_) or relative total tissue hemoglobin (rTHb) identified dysfunctional autoregulation when ABP was below the laser Doppler flow-defined LLA.

Useful monitoring methods must have high signal-to-noise ratios. Many of the most commonly used NIRS autoregulation methods, including the correlation HVx and COx indices, generate high signal variability that may not reflect physiologic processes. This has limited translation of the correlation indices into pediatric clinical practice. In this study, we found that all wavelet indices had lower variability than their corresponding correlation index. The wavelet methodology decreases signal noise by using a coherence threshold to eliminate poorly coherent signals. This increases the likelihood of analyzing physiologic relevant phase shifts between blood pressure and cerebral blood volume or cerebral blood flow instead of signal noise.

The ability to distinguish ABP above from that below the LLA did not differ between COx and wCOx or between PRx and wPRx. We previously published that wPRx predicts patient outcome better than PRx in a clinical study of adult traumatic brain injury^10^. The absence of a difference between wavelet and correlation PRx and COx in our piglet study may be related to different preclinical and clinical autoregulation monitoring conditions. In piglets, we generate distinct time periods with several hours of functional or dysfunctional autoregulation alongside constant normocapnea, constant oxygen supply, minimal change in hemoglobin level, and a steady sedation regimen. These experimental conditions optimize autoregulation monitoring. In contrast, brain injured patients have greater clinical physiologic variability which could increase signal noise in the correlation indices. The lower signal variability from the wavelet method could therefore result in better clinical performance^10^ yet also show equivalent performance in our experimental model which is already designed to minimize signal noise.

However, HVx had significantly higher discriminatory value than wHVx in detecting dysfunctional autoregulation. Nonetheless, both indices accurately distinguished ABP above the LLA from that below the LLA with an HVx AUC of 0.95 and a wHVx AUC of 0.87. Clinical studies that compare wavelet to correlation indices are needed to clarify how these metrics perform in children recovering from cardiac arrest and hypoxic brain injury.

We induced severe hypotension to intentionally impair autoregulatory function in piglets. Because extremely low blood pressure is avoided clinically, the data with ABP above the LLA to identify ABPopt is highly clinically relevant. The wavelet indices generated clearer U-shaped curves with ABPopt at the vertex than did the correlation indices. This difference was most apparent when blood pressure rose above ABPopt (Fig. 4). ABPopt can be identified by different methods, including techniques that require neuromonitoring software or visual inspection of the index curves. Because many investigators and clinicians do not have access to the multi-window approach, we also graphed the index curves to look for a U-shape and ABPopt at the vertex in concordance with the methodology of some pediatric clinical studies^4,7,9^. Whether identifying ABPopt using wavelet NIRS improves neurologic outcome after pediatric cardiac arrest deserves study.

Threshold index values for distinguishing functional from dysfunctional autoregulation or vasoreactivity in clinical research often rely on preclinical models. In our piglet model of pediatric cardiac arrest and hypotension, maximal sensitivity and specificity for distinguishing blood pressure above from below the LLA was at wCOx of 0.26 and wHVx of 0.19. Because these threshold values may only be relevant during extreme hypotension, associations between fine neurologic outcomes and autoregulation might be best identified using blood pressure deviation from ABP_OPT_.

It is important to distinguish the wavelet indices’ low signal variation from low physiologic variability, such as the association between low heart rate variability and brain injury in neonatal hypoxia^20^. We calculated wavelet and correlation indices in the same piglets during identical physiologic changes from induced hypotension. Clinical neonatal wavelet NIRS methods have also been studied in hypoxic-ischemic encephalopathy^21,22^ and prematurity^23^.

Our finding that wPRx and PRx as well as wCOx and COx had similar ability to identify dysfunctional autoregulation contrasts to another study from our research group that showed greater accuracy from wPRx than PRx in piglets with ventricular artificial cerebrospinal fluid (aCSF) infusions^24^. The current study’s piglets had mean ICP ≤ 15 mmHg related to post-cardiac arrest, global hypoxic brain injury^14,18,19^. The aCSF infusions generated ICP levels of approximately 10 and 20 mmHg and mimicked hydrocephalus^24^. Our whole body hypoxic-asphxyic cardiac arrest protocol generates acute global vasogenic and cytotoxic cerebral edema, oxidative stress^25^, inflammation^25^, and neural cell death^19,26^ in the same brain region with NIRS monitoring. Further studies are needed to explore and compare the accuracy of different autoregulation monitoring techniques in different types of brain injury.

Some of the piglets received whole-body hypothermia. Therapeutic hypothermia is the standard of clinical care for neonatal hypoxic-ischemic encephalopathy, including after perinatal cardiac arrest. Though the Therapeutic Hypothermia After Pediatric Cardiac Arrest clinical trial showed unclear benefit from hypothermia^27^, the delay in inducing hypothermia may have reduced therapeutic potential in this trial. Accordingly, some investigators continue to study early induction of hypothermia or hypothermia with adjuvant treatments for pediatric cardiac arrest. We did not have sufficient sample size to stratify the piglets according to temperature treatment. Nonetheless, our primary research question was to test agreement between wavelet and correlation indices in paired analyses. Moreover, our data suggest that wavelet NIRS methodology could be tested during hypothermic cardiopulmonary bypass or during therapeutic hypothermia for neonatal hypoxic-ischemic encephalopathy or adult cardiac arrest in future studies.

## Limitations

We reanalyzed piglet cohorts from past studies^14,18,19^, and we only studied males. Nonetheless, the large preclinical sample size of 68 piglets provided an opportunity to test and validate wavelet methodology in a developing brain, cardiac arrest model without sacrificing additional animals. Further research is needed to test the accuracy of wavelet NIRS methodology in both sexes, in prospective studies, and after longer recovery from cardiac arrest as secondary brain injury evolves with and without vasopressor treatment. We did not assess the piglets’ upper limit of autoregulation because cardiopulmonary failure often occurs before this limit can be reached after piglet cardiac arrest^14,18^.

## Conclusion

We found that wavelet NIRS indices may be useful tools to monitor cerebrovascular autoregulation in the developing brain after cardiac arrest. Wavelet methodology had low signal variability, accurately distinguished functional from dysfunctional autoregulation, identified ABPopt, and generated a clear U-shaped curve with visually apparent ABPopt at the vertex. Further studies are needed to evaluate the potential of wavelet NIRS autoregulation monitoring after pediatric cardiac arrest.

## Materials and Methods

We reanalyzed data from three previously published piglet studies of cardiac arrest and hypothermia^14,18,19^ to conserve animals. All procedures were approved by the Animal Care and Use Committee at Johns Hopkins University, and animal care was in accord with the National Institutes of Health guidelines. In the first study, piglets were randomized to cardiac arrest or sham procedure followed by a 6-h recovery period with whole body hypothermia or normothermia^18^. In the second study, piglets were randomized to cardiac arrest or sham procedure followed by overnight hypothermia with or without rewarming^14^. In the third study, piglets were randomized to cardiac arrest or sham procedure with 1 or 2 days of normothermic recovery^19^. Though we also published these piglets’ data for a coherence-conditioned phase autoregulation technique^28^, the current study is a unique analysis that includes *de novo* LLA calculations, application of wavelet semblance methodology, and identification of ABPopt using a multi-window technique in 68 neonatal piglets resuscitated from cardiac arrest.

### Animal experiments

We previously published detailed methods for the piglet experiments^14,18,19^. Briefly, male piglets (3–5 days old, 1–2.5 kg) were anesthetized for intubation and placement of femoral arterial and venous cannulae, an intraventricular ICP monitor, and a cortical laser Doppler flow probe (Moor Instruments, Devon, U.K.; model DRT4; 60 Hz) to measure red blood cell flux. The laser Doppler was positioned 5 mm from the ICP monitor. A pediatric NIRS probe (Covidien, Boulder, CO) was placed over the pig’s contralateral frontoparietal cortex. The NIRS probe’s light-emitting diodes had shallow and deep photodiode distances of 30 and 40 mm, respectively. A 5-French balloon catheter was placed in the inferior vena cava for later inflation to induce hypotension. The anesthetic consisted of 50%/50% nitrous oxide/oxygen, isoflurane 1.5-2%, and fentanyl (10 µg/kg bolus followed by 10–20 µg/kg/h, intravenous). The isoflurane was decreased to 0.4–0.8% upon completion of surgery. The piglets were mechanically ventilated to maintain normocapnea.

The piglets were randomized to receive hypoxic-asphyxic cardiac arrest or sham procedure. The cardiac arrest protocol consisted of decreasing the fractional inspired concentration of oxygen (FiO_2_) to 10% for 45 minutes followed by 5 minutes of room air. Then, the endotracheal tube was clamped to produce 7 minutes of asphyxia. The piglets were resuscitated with chest compressions, 100% oxygen, and epinephrine (100 µg/kg, intravenous). This model produces robust cortical and white matter injury^19,26,29,30^. Sham piglets received the same surgery but without hypoxia-asphyxia. After resuscitation or time equivalent in shams, the FiO_2_ was decreased to 50% for the remainder of the experiment. The piglets were additionally randomized to normothermia (goal 38.0–39.0, which is normothermic for swine), sustained whole body hypothermia to 32 °C, or hypothermia with rewarming and up to 2-day recovery as previously described^14,18,19^.

Methods for signal sampling, induced hypotension, and LLA calculations were identical among all piglets. Laser Doppler flow, ICP, NIRS, and ABP data were sampled at 100 Hz with ICM+ software (University of Cambridge, Cambridge Enterprise, Cambridge, UK). The rTHb was measured by NIRS at the 805 nm wavelength, which is isosbestic to both oxy- and deoxyhemoglobin. The rTHb is a surrogate measure of cerebral blood volume during autoregulatory vasoreactivity^31^. The laser Doppler flow, ICP, NIRS, and ABP data were averaged in consecutive 10-second means to remove high-frequency waveforms from pulse and respiration. The remaining data represent the slow wave oscillations that occur during autoregulatory vasoreactivity^31^.

### LLA determination

We slowly decreased the piglet’s blood pressure over approximately 2–3 hours by inflating the balloon catheter in the inferior vena cava. Each piglet’s ABP at the LLA was identified by ICM+ software, which uses piecewise regression to dichotomize the data and fit two linear regression lines with the lowest combined error squared. The LLA is at the intersection of these two lines^24^. We considered each piglet’s laser Doppler flow-derived LLA to be the “gold standard” to test whether the autoregulation indices identified ABP above or ABP below the LLA.

### Correlation vasoreactivity and autoregulation indices

We calculated a moving Pearson correlation coefficient from 30 paired samples in consecutive, 300-second moving windows to generate each index. The hemoglobin volume reactivity index (HVx) was calculated as the correlation from ABP and NIRS rTHb. Functional autoregulatory vasoreactivity is indicated by negative or near-zero HVx values because ABP and cerebral blood volume are negatively or not correlated. When blood pressure is below the LLA, HVx becomes increasingly positive and approaches +1 with progressive impairments in vasoreactivity when ABP and cerebral blood volume correlate^31^.

We calculated the cerebral oximetry autoregulation index (COx) from ABP and rSO_2_ using analogous methods^13^. Negative or near-zero COx values indicate functional autoregulation. When blood pressure progressively decreases to below the LLA, ABP and cerebral blood flow positively correlate to generate an increasingly positive COx index that approaches +1.

The pressure reactivity index (PRx) was calculated as the correlation between ABP and ICP. Low PRx indicates functional autoregulatory vasoreactivity, and high PRx indicates dysfunctional vasoreactivity.

### Wavelet transform phase indices

We calculated wavelet transform phase between ABP and NIRS rTHb using a complex, continuous wavelet transform^10,16^ in the low-frequency range of 0.007–0.05 Hz. Intracranial slow waves from autoregulatory vasoreactivity are known to occur in this frequency range^31^. Briefly, the wavelet transform phase at each scale-frequency point was calculated from 800-second moving windows of data that were updated every 10 seconds. Due to the edge effect, approximately 500 seconds of data were rejected to leave 300 seconds of data for analysis. This resulted in wavelet analysis in 300-second windows, which was identical to the window length used for the correlation indices. Then, we calculated the wavelet semblance^32^, the cosine of wavelet phase shift, to generate the wavelet hemoglobin volume reactivity index (wHVx). We used a wavelet coherence threshold of <0.46 to reject corresponding unreliable phase values (the threshold of 0.46 was decided by Monte Carlo simulations and more details can be found from our previous publication^33^). The cosine operation accounts for phase wrapping because the obtained value is equivalent to the normalized real part of the wavelet transfer function and is not subject to wrapping effects. Finally, we averaged the values along the frequency domain to obtain one wHVx value for each time point.

The wHVx index is a continuous value that ranges from −1 to +1. A wHVx of –1 indicates a 180° phase shift with complete inverse correlation between ABP and rTHb. No correlation generates a wHVx of zero. The wHVx is +1 with 0° phase shift and complete positive correlation between ABP and rTHb (e.g., complete impairment in vasoreactivity with full pressure passivity). Therefore, the directionality of wHVx is the same as that for HVx.

Using analogous methods, we calculated wCOx as the wavelet semblance between ABP and rSO_2_ and wPRx as the wavelet semblance between ABP and ICP. Functional autoregulation generates negative or near-zero wCOx and wPRx, whereas impaired autoregulation generates positive wCOx and wPRx.

### Calculation of ABPopt

**Figure 5:**
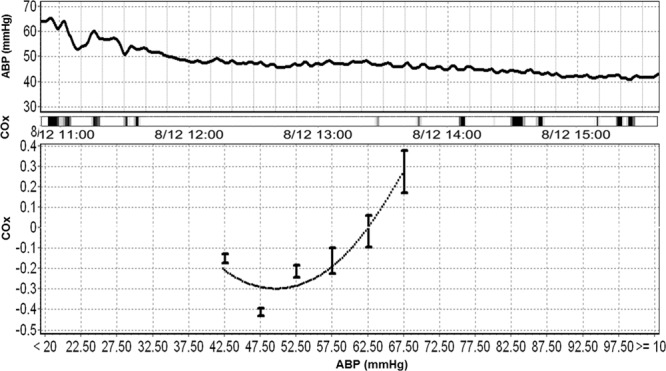
Calculating the optimal mean arterial blood pressure (ABPopt) in a piglet using the cerebral oximetry index (COx). Average COx values were sorted into their respective 5-mmHg bins of ABP to generate a U-shaped index curve. The ABPopt is located at the U-shaped curve’s vertex. This piglet’s ABPopt was 49.5 mmHg.

We identified each piglet’s ABPopt with most functional vasoreactivity (HVx, wHVx, PRx, wPRx) or most functional autoregulation (COx, wCOx) when blood pressure exceeded the LLA. Briefly, the ICM+ software generated median ABP values from 5-minute windows across time alongside the index. We set the ABP upper and lower limits for ABPopt calculation at 20 and 100 mmHg. Average index values were sorted into their corresponding 5-mmHg bins of ABP. The mean and standard error of each index (y-axis) were plotted against the 5-mmHg bins’ mean ABP (x-axis) to generate an error bar chart. The index data were Fisher transformed to achieve normal distribution and thereby eliminate the ceiling effect from having a maximum index value of 1. Then, the software applied an automatic curve-fitting method to determine the 5-mmHg bin of ABPopt where the index was lowest. The curve-fitting error was calculated as the square root of the average sum of the squared differences between the 5-mmHg bin averaged index data and the fitted values. Theoretically, this process should generate a smooth U-shaped curve with the ABPopt at the lowest point (vertex; Fig. 5).

We then applied our multi-window approach, which we previously described in detail^34^. Each ABPopt was calculated from twelve 2–4-hour windows that were updated every 10 minutes to generate 12 index-ABP plots. The plots were then given a combined weight factor according to window duration, curve-fit error, and curve shape. Shorter windows of data and data with smaller curve-fit errors were weighted more heavily and thus contributed more to the ABPopt calculation. Data with fitted curves that did not form a U-shape and that did not have a vertex received less weight and contributed less to the ABPopt calculation. Each piglet’s final ABPopt was computed as the weighted average of the 12 plots from HVx, COx, PRx, wHVx, wCOx, and wPRx when ABP exceeded the LLA.

### Statistical analysis

Statistical analyses were performed and graphs were generated with SPSS (version 25, IBM, Armonk, NY, USA). The ICM+ software generated more than 500 NIRS index values across changes in blood pressure for each piglet. To analyze whether each NIRS index distinguished blood pressure above the laser Doppler flow-derived LLA from that below the LLA, we analyzed one mean index value for all blood pressure levels above the LLA and one mean index value for all blood pressures below the LLA. For example, this process generated one wHVx value for ABP above the LLA and one wHVx value for ABP below the LLA for each pig. We conducted paired (within pig) comparisons of HVx and wHVx, PRx and wPRx, and COx and wCOx using Pearson correlations.

The ICM+ software also calculated each index’s standard deviation (SD) from more than 300 index values when blood pressure exceeded the LLA for each pig. This calculation generated one SD for each index per piglet, and we compared the SDs between wavelet and correlation indices using paired t-tests. We report index variability as each index’s mean SD ± the SD of all the piglets’ SDs.

In addition, we tested the indices’ sensitivity and specificity in distinguishing ABP above the LLA from that below the LLA using paired receiver operator characteristic (ROC) curves. Differences between ROC areas under the curve (AUC) were tested using the DeLong’s test for two correlated ROC curves^35^.

ABPopt values from the wavelet- and correlation-derived indices were compared by Pearson correlations and Bland-Altman plots. We also graphed the indices by blood pressure relative to each piglet’s ABPopt in a descriptive evaluation of the data.

Because *a priori* data about wavelet NIRS monitoring in the developing brain after cardiac arrest were not available, we could not conduct sample size or power calculations. We will use the findings from the current project to inform future research.

## Supplementary information

Supplementary information.

## Data Availability

The data is available to share on reasonable request to liuxiuyun1@gmail.com. The minimal data set which can be used to reach the conclusions are deposited publicly in figshare as below: ‘ Liu, Xiuyun (2019), Piglet data for wavelet analysis, figshare, https://figshare.com/articles/Piglet_data_for_wavelet_analysis/9936470 ’.

## References

[1] Iordanova, B., Li, L., Clark, R. S. B. & Manole, M. D. Alterations in Cerebral Blood Flow after Resuscitation from Cardiac Arrest. *Front. Pediatr*. 10.3389/fped.2017.00174. (2017). 10.3389/fped.2017.00174 PMC5561008 28861407

[2] Pham, P., Bindra, J., Chuan, A., Jaeger, M. & Aneman, A. Are changes in cerebrovascular autoregulation following cardiac arrest associated with neurological outcome? Results of a pilot study. *Resuscitation*. 10.1016/j.resuscitation.2015.08.007 (2015). 10.1016/j.resuscitation.2015.08.007 26316278

[3] Sekhon, M. S. *et al*. Using the relationship between brain tissue regional saturation of oxygen and mean arterial pressure to determine the optimal mean arterial pressure in patients following cardiac arrest: A pilot proof-of-concept study. *Resuscitation*. 10.1016/j.resuscitation.2016.05.019 (2016). 10.1016/j.resuscitation.2016.05.019 27255957

[4] Lee, J. K. *et al*. A pilot study of cerebrovascular reactivity autoregulation after pediatric cardiac arrest. *Resuscitation*. 10.1016/j.resuscitation.2014.07.006 (2014). 10.1016/j.resuscitation.2014.07.006 PMC4164565 25046743

[5] Aries MJH (2012). Continuous determination of optimal cerebral perfusion pressure in traumatic brain injury. Crit. Care Med..

[6] Brady KM (2009). The lower limit of cerebral blood flow autoregulation is increased with elevated intracranial pressure. Anesth. Analg..

[7] Burton, V. J. *et al*. A pilot cohort study of cerebral autoregulation and 2-year neurodevelopmental outcomes in neonates with hypoxic-ischemic encephalopathy who received therapeutic hypothermia. *BMC Neurol*. 10.1186/s12883-015-0464-4 (2015). 10.1186/s12883-015-0464-4 PMC4618147 26486728

[8] Tekes, A. *et al*. Apparent diffusion coefficient scalars correlate with near-Infrared spectroscopy markers of cerebrovascular autoregulation in neonates cooled for perinatal hypoxic-Ischemic injury. *Am. J. Neuroradiol*. 10.3174/ajnr.A4083 (2015). 10.3174/ajnr.A4083 PMC4359612 25169927

[9] Lee JK (2017). Optimizing Cerebral Autoregulation May Decrease Neonatal Regional Hypoxic-Ischemic Brain Injury. in. Developmental Neuroscience.

[10] Liu X (2017). Cerebrovascular pressure reactivity monitoring using wavelet analysis in traumatic brain injury patients: A retrospective study. PLOS Med..

[11] Sekhon, M. S. *et al*. The Burden of Brain Hypoxia and Optimal Mean Arterial Pressure in Patients With Hypoxic Ischemic Brain Injury After Cardiac Arrest. *Crit. Care Med*. 1. 10.1097/CCM.0000000000003745 (2019). 10.1097/CCM.0000000000003745 30889022

[12] Da Costa CS (2015). Monitoring of cerebrovascular reactivity for determination of optimal blood pressure in preterm infants. J. Pediatr..

[13] Lee, J. K., Williams, M., Reyes, M. & Ahn, E. S. Cerebrovascular blood pressure autoregulation monitoring and postoperative transient ischemic attack in pediatric moyamoya vasculopathy. *Paediatr. Anaesth*. 10.1111/pan.13293 (2018). 10.1111/pan.13293 PMC5960234 29205668

[14] Larson, A. C. *et al*. Cerebrovascular autoregulation after rewarming from hypothermia in a neonatal swine model of asphyxic brain injury. *J. Appl. Physiol*.. 10.1152/japplphysiol.00238.2013 (2013). 10.1152/japplphysiol.00238.2013 PMC3841826 24009008

[15] Ono, M. *et al*. Cerebral blood flow autoregulation is preserved after hypothermic circulatory arrest. *Ann. Thorac. Surg*. 10.1016/j.athoracsur.2013.07.086 (2013). 10.1016/j.athoracsur.2013.07.086 PMC3972490 24446562

[16] Liu X (2018). Wavelet pressure reactivity index: A validation study. J. Physiol..

[17] Czosnyka M. *et al*. Continuous assessment of the cerebral vasomotor reactivity in head injury. *Neurosurgery*. **41**(1), 11–7 (1997). 10.1097/00006123-199707000-00005 9218290

[18] Lee JK (2011). Cerebral blood flow and cerebrovascular autoregulation in a swine model of pediatric cardiac arrest and hypothermia. Crit Care Med.

[19] Lee, J. K. *et al*. Noninvasive autoregulation monitoring in a swine model of pediatric cardiac arrest. *Anesth. Analg*. 10.1213/ANE.0b013e31824762d5 (2012). 10.1213/ANE.0b013e31824762d5 PMC3310318 22314692

[20] Metzler, M. *et al*. Pattern of brain injury and depressed heart rate variability in newborns with hypoxic ischemic encephalopathy. *Pediatr. Res*. 10.1038/pr.2017.94 (2017). 10.1038/pr.2017.94 PMC5570625 28376079

[21] Chalak LF, Zhang R (2017). New Wavelet Neurovascular Bundle for Bedside Evaluation of Cerebral Autoregulation and Neurovascular Coupling in Newborns with Hypoxic-Ischemic Encephalopathy. Dev. Neurosci..

[22] Mitra, S. *et al*. Pressure passivity of cerebral mitochondrial metabolism is associated with poor outcome following perinatal hypoxic ischemic brain injury. *J. Cereb. Blood Flow Metab*. 10.1177/0271678X17733639 (2019). 10.1177/0271678X17733639 PMC6311664 28949271

[23] Beausoleil, T. P., Janaillac, M., Barrington, K. J., Lapointe, A. & Dehaes, M. Cerebral oxygen saturation and peripheral perfusion in the extremely premature infant with intraventricular and/or pulmonary haemorrhage early in life. *Sci. Rep*. 10.1038/s41598-018-24836-8 (2018). 10.1038/s41598-018-24836-8 PMC5916916 29695729

[24] Liu, X. *et al*. Wavelet pressure reactivity index: a validation study. *Apollo - Univ. Cambridge Repos*. 10.17863/cam.33006 (2018). 10.1113/JP274708 PMC6046066 29665012

[25] Bhalala, U. S., Koehler, R. C. & Kannan, S. Neuroinflammation and neuroimmune dysregulation after acute hypoxic-ischemic injury of developing brain. *Frontiers in Pediatrics*. 10.3389/fped.2014.00144 (2015). 10.3389/fped.2014.00144 PMC4294124 25642419

[26] Wang, B. *et al*. Rewarming from therapeutic hypothermia induces cortical neuron apoptosis in a swine model of neonatal hypoxic-ischemic encephalopathy. *J. Cereb. Blood Flow Metab*. 10.1038/jcbfm.2014.245 (2015). 10.1038/jcbfm.2014.245 PMC4420851 25564240

[27] Moler F (2017). Therapeutic Hypothermia after In-Hospital Cardiac Arrest in Children. N Engl J Med.

[28] Govindan, R. B. *et al*. Comparison of Frequency- and Time-Domain Autoregulation and Vasoreactivity Indices in a Piglet Model of Hypoxia-Ischemia and Hypothermia. *Dev. Neurosci*. 10.1159/000499425 (2019). 10.1159/000499425 PMC6824917 31048593

[29] Wang, B. *et al*. White matter apoptosis is increased by delayed hypothermia and rewarming in a neonatal piglet model of hypoxic ischemic encephalopathy. *Neuroscience*10.1016/j.neuroscience.2015.12.046 (2016). 10.1016/j.neuroscience.2015.12.046 PMC4724504 26739327

[30] Santos, P. T. *et al*. Proteasome biology is compromised in white matter after asphyxic cardiac arrest in neonatal piglets. *J. Am. Heart Assoc*. 10.1161/JAHA.118.009415 (2018). 10.1161/JAHA.118.009415 PMC6474957 30371275

[31] Lee JK (2009). Cerebrovascular reactivity measured by near-infrared spectroscopy. Stroke.

[32] Addison, P. S. *The Illustrated Wavelet Transform Handbook: Introductory Theory and Applications in Science, Engineering, Medicine and Finance 1st Edition*. *Introductory Theory and Applications in Science, Engineering, Medicine and Finance Napier University, Edinburgh, UK* (CRC Press, 2002).

[33] Liu, X. *et al*. Wavelet pressure reactivity index: a validation study. *J. Physiol*. 10.1113/JP274708 (2018). 10.1113/JP274708 PMC6046066 29665012

[34] Liu X (2017). Monitoring of optimal cerebral perfusion pressure in traumatic brain injured patients using a multi-window weighting algorithm. J Neurotrauma.

[35] ER, D., DM, D. & DL, C.-P. Comparing the areas under two or more correlated receiver operating characteristic curves: a nonparametric approach. *Biometrics* (1988). 3203132

