# Author Correction: Comparison of wavelet and correlation indices of cerebral autoregulation in a pediatric swine model of cardiac arrest

**DOI:** 10.1038/s41598-021-01918-8

**Published:** 2021-11-12

**Authors:** Xiuyun Liu, Xiao Hu, Ken M. Brady, Raymond Koehler, Peter Smielewski, Marek Czosnyka, Joseph Donnelly, Jennifer K. Lee

**Affiliations:** 1 Department of Anesthesiology and Critical Care Medicine, School of Medicine, Johns Hopkins University, Baltimore, MD USA; 2 Department of Physiological Nursing, University of California, San Francisco, CA USA; 3 Department of Neurosurgery, School of Medicine, University of California, Los Angeles, CA USA; 4 Department of Neurological Surgery, University of California, San Francisco, CA USA; 5 Institute of Computational Health Sciences, University of California, San Francisco, CA USA; 6 Department of Anesthesiology, Ann & Robert H. Lurie Children’s Hospital of Chicago, Northwestern University, Chicago, IL USA; 7 Brain Physics Laboratory, Department of Clinical Neurosciences, Addenbrooke’s Hospital, University of Cambridge, Cambridge, UK; 8 Department of Anaesthesiology, University of Auckland, Auckland, New Zealand; 9 Division of Pediatric Anesthesiology, Department of Anesthesiology and Critical Care Medicine, Johns Hopkins University, Baltimore, MD USA; 10 Institute of Electronic Systems, Warsaw University of Technology, Warsaw, Poland

Correction to: *Scientific Reports* https://doi.org/10.1038/s41598-020-62435-8, published online 03 April 2020

The original version of this Article contained errors. Figure 1a duplicated Figure 1c. The figure legend was also revised for accuracy and “squares” now reads “dots”.

The original Figure 1 and accompanying legend appear below.

**Figure 1:**
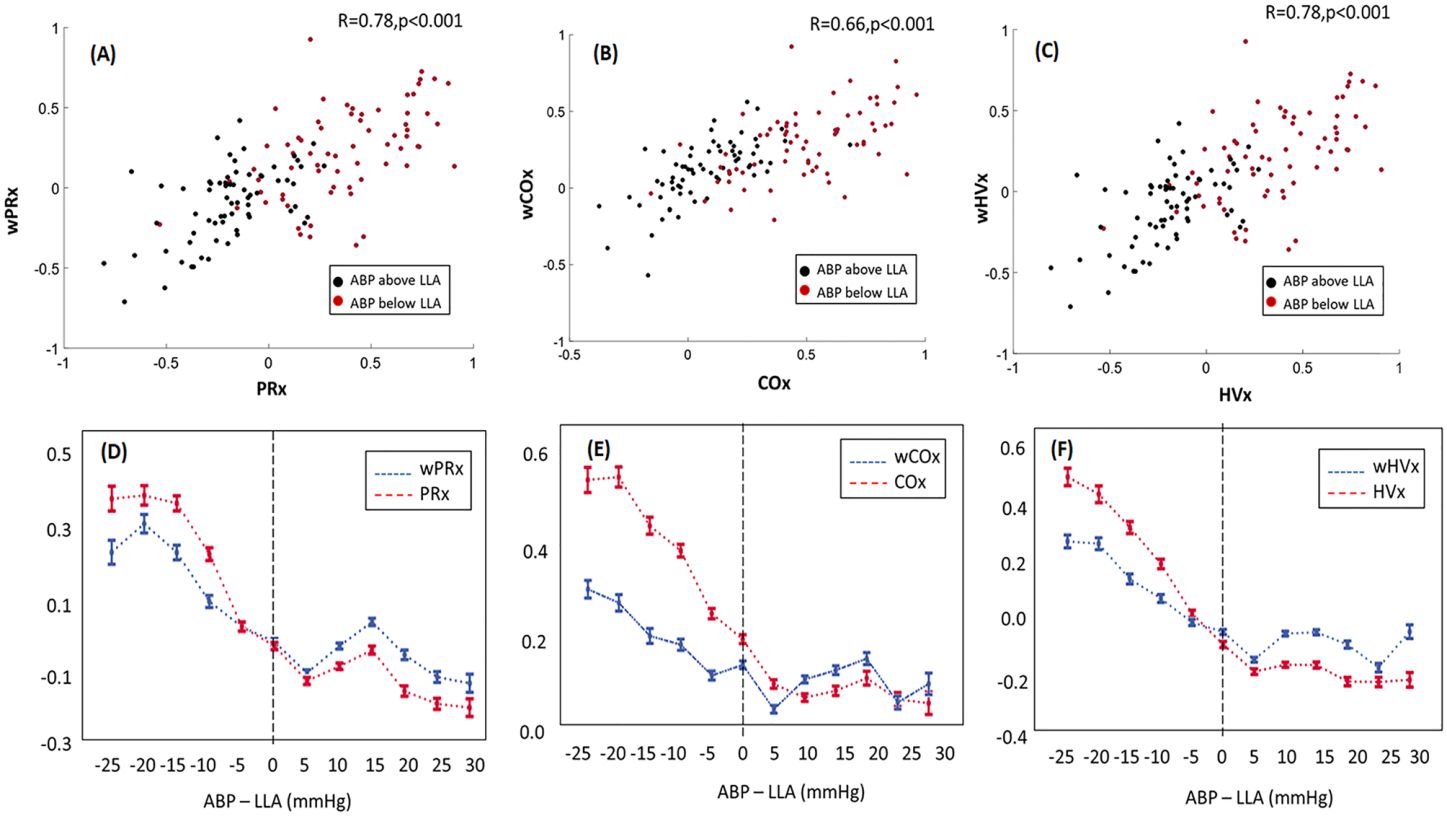
Both wavelet and correlation indices increased while mean arterial blood pressure (ABP) decreased below the lower limit of autoregulation (LLA). (**A**–**C**) In paired comparisons among 68 piglets, the wavelet autoregulation indices (wPRx, wCOx, wHVx) and correlation indices (PRx, COx and HVx) correlated with each other. Each piglet provided one index value averaged from ABP above the LLA and one index from ABP below the LLA, thereby generating 136 data points per graph. The black squares are index values when ABP exceeded the LLA. The red dots are index values when ABP was below the LLA. (**D**–**F**) Graphical depiction of the indices across changes in blood pressure. Each piglet’s ABP LLA is centered at zero on the x-axis (dashed line) to show the wavelet method (blue lines) and correlation method (red lines) responses to changes in blood pressure. Data are shown as mean ± standard error of the mean.

The data from the corrected Figure 1 was re-analysed; the Results section, under the subheading ‘Distinguishing ABP above from ABP below the LLA’, was revised as follows to reflect this:

“Wavelet indices and their respective correlation indices were highly correlated (r = 0.78, p < 0.001 for PRx and wPRx; r = 0.66, p < 0.001 for COx and wCOx; and r = 0.78, p = 0.002 for HVx and wHVx; and; Figure 1A–C).”

now reads:

“Wavelet indices and their respective correlation indices were highly correlated (r = 0.78, p < 0.001 for PRx and wPRx; r = 0.66, p < 0.001 for COx and wCOx; and r = 0.69, p < 0.001 for HVx and wHVx; Figures 1A–1C).”

The original Article has been corrected. This correction does not affect the conclusions of the Article.

