# Supplementary material for: Comparison of wavelet and correlation indices of cerebral autoregulation in a pediatric swine model of cardiac arrest
Source: Sci Rep. 2020 Apr 3;10:5926. doi: 10.1038/s41598-020-62435-8 (PMC7125097; doi:10.1038/s41598-020-62435-8)
Supplement: Supplementary file 1 — Supplementary information. [file 41598_2020_62435_MOESM1_ESM.pdf]

Comparison of wavelet and correlation indices of cerebral autoregulation in a pediatric swine model of cardiac arrest

Authors: Xiuyun Liu, PhD;Xiao Hu, PhD; Ken M. Brady, MD; Raymond Koehler, PhD; Peter Smielewski, PhD; Marek Czosnyka, PhD; Joseph Donnelly, MBChB; Jennifer K. Lee, MD

Supplemental Figure S1

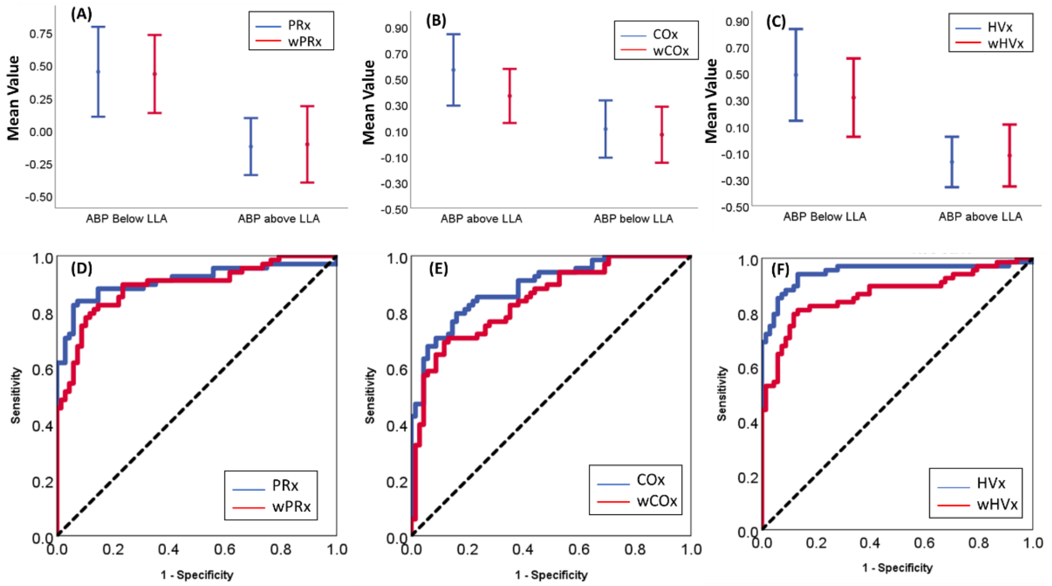

S1 Figure. (A)-(C) Paired comparisons of average indices for ABP above or below the lower limit of autoregulation (LLA). The indices were higher when the arterial blood pressure (ABP) was below LLA than when ABP was above the LLA. The data are shown as means with SD. (D)-(F) Areas under the receiver operator characteristic curve for each index's ability to distinguish ABP above versus below the LLA.
